# Context-dependent memory decay is evidence of effort minimization in motor learning: a computational study

**DOI:** 10.3389/fncom.2015.00004

**Published:** 2015-02-04

**Authors:** Ken Takiyama

**Affiliations:** Brain Science Institute, Tamagawa UniversityTokyo, Japan

**Keywords:** motor learning, neural network modeling, context-dependent memory decay, effort minimization, motor primitive

## Abstract

Recent theoretical models suggest that motor learning includes at least two processes: error minimization and memory decay. While learning a novel movement, a motor memory of the movement is gradually formed to minimize the movement error between the desired and actual movements in each training trial, but the memory is slightly forgotten in each trial. The learning effects of error minimization trained with a certain movement are partially available in other non-trained movements, and this transfer of the learning effect can be reproduced by certain theoretical frameworks. Although most theoretical frameworks have assumed that a motor memory trained with a certain movement decays at the same speed during performing the trained movement as non-trained movements, a recent study reported that the motor memory decays faster during performing the trained movement than non-trained movements, i.e., the decay rate of motor memory is movement or context dependent. Although motor learning has been successfully modeled based on an optimization framework, e.g., movement error minimization, the type of optimization that can lead to context-dependent memory decay is unclear. Thus, context-dependent memory decay raises the question of what is optimized in motor learning. To reproduce context-dependent memory decay, I extend a motor primitive framework. Specifically, I introduce motor effort optimization into the framework because some previous studies have reported the existence of effort optimization in motor learning processes and no conventional motor primitive model has yet considered the optimization. Here, I analytically and numerically revealed that context-dependent decay is a result of motor effort optimization. My analyses suggest that context-dependent decay is not merely memory decay but is evidence of motor effort optimization in motor learning.

## 1. Introduction

After a few years of not training in a previously learned sport, it is easy to forget how to move one's body in a manner that is suitable for the sport. This phenomenon is known as memory decay and is a well-known component of motor learning. Indeed, recent studies suggest that motor learning includes at least two processes: the minimization of movement error and motor memory decay (Scheidt et al., 2001; Smith et al., 2006; Hirashima and Nozaki, 2012).

The properties of error minimization have been extensively investigated using perturbation paradigms such as a force field paradigm (Shadmehr and Mussa-Ivaldi, 1994) or a visuomotor transformation paradigm (Krakauer et al., 1999). In these paradigms, subjects exhibit trial-by-trial adaptations to the novel environment, and the movement error between the desired and actual movements decreases in each trial. The learning effect of error minimization generalizes when kinematics (e.g., the movement direction) change (Thoroughman and Shadmehr, 2000; Donchin et al., 2003). For example, when trained in unimanual reaching movements toward a target direction θ, the learning effects of the error minimization are partially available for reaching movements toward other target directions. In other words, the availability of the learning effect depends on context (in this example, the context is equal to the target direction). In the following, I refer to the learning effects of error minimization as learning effects and to the context-dependent availability of learning effects as generalization.

Generalization can be modeled using a motor primitive framework (Thoroughman and Shadmehr, 2000; Donchin et al., 2003). In this framework, the activities of the motor primitive determine the motor commands, the recruitment pattern of the motor primitive is determined by a target direction, and the motor commands are updated to minimize movement error. The motor primitive framework can naturally reproduce the generalization because a group of motor primitives recruited in reaching movements toward a target direction θ overlaps with a group of primitives recruited in movements toward other directions, θ′(≠ θ), which results in learning effects embedded in the motor primitives responsible for reaching movements toward θ are partially available in other reaching movements. Recent studies have revealed that the motor primitive framework can reproduce generalization in various situations, such as motor learning in a force field (Thoroughman and Shadmehr, 2000; Donchin et al., 2003) or visuomotor rotation (Tanaka et al., 2009), motor learning of bimanual reaching movements (Yokoi et al., 2011), motor learning of unimanual reaching movements when the both target direction and shoulder posture change (Brayanov et al., 2012), the dependence of the generalization on error-feedback information (Taylor et al., 2012), and the generalization between reaching movements of the right hand and those of the left hand (Yokoi et al., 2014). Thus, the properties of error minimization or generalization in a wide range of situations can be explained by the motor primitive framework.

By contrast, the properties of memory decay have rarely been investigated. In the perturbation paradigms, the memory of the learning effects decays in each trial (Scheidt et al., 2000). In the following, I refer to the memory of learning effects as motor memory. Nearly all the models of motor primitives assume that the rate of motor memory decay is context independent (Thoroughman and Shadmehr, 2000; Scheidt et al., 2001; Donchin et al., 2003; Smith et al., 2006; Tanaka et al., 2009; Yokoi et al., 2011; Brayanov et al., 2012; Hirashima and Nozaki, 2012; Taylor et al., 2012; Takiyama and Okada, 2012a; Yokoi et al., 2014), i.e., the motor memory learned with reaching movements toward θ decays during the trials of the reaching movements at the same speed as during trials of reaching movements toward other directions, θ′(≠ θ). In the motor primitive framework, the context-independent decay is equivalent to weight decay (Hirashima and Nozaki, 2012). The weight values of each motor primitive determine how the primitives contribute to generate motor commands, and the weight decay introduces a trial-by-trial decay of the magnitudes of the weight values (see the Weight Decay section in the Results for details). However, a recent behavioral experiment found that the decay rate of motor memory can be context dependent (Ingram et al., 2013). When trained in reaching movements toward a target direction θ, the motor memory decayed faster during the trials of reaching movements toward θ than during trials of reaching movements toward other target directions θ′(≠ θ). The authors of this study reported that a conventional model of motor learning could be fit well to their data with the assumptions of both generalization function and context-dependent memory decay. Notably, the authors heuristically introduced context-dependent decay to fit the conventional model to the actual data. Thus, the reason that memory decay is context dependent remains unclear. Although motor learning has conventionally been modeled as an optimization framework (Thoroughman and Shadmehr, 2000; Scheidt et al., 2001; Donchin et al., 2003; Smith et al., 2006; Tanaka et al., 2009; Yokoi et al., 2011; Brayanov et al., 2012; Hirashima and Nozaki, 2012; Taylor et al., 2012; Takiyama and Okada, 2012a,b; Yokoi et al., 2014), e.g., movement error minimization, no conventional optimization framework can reproduce the context-dependent memory decay, i.e., context-dependent memory decay raises the question of what is optimized in motor learning.

In the current study, I extend a motor primitive framework to reproduce the context-dependent decay of motor memory. Specifically, I introduce effort minimization, i.e., the minimization of motor command magnitude (Harris and Wolpert, 1998; Fagg et al., 2002), into the framework because previous studies have reported the minimization during motor learning processes, such as adapting to a force field (Emken et al., 2007; Huang et al., 2012) and a visuomotor transformation (Huang and Ahmed, 2014) and because no conventional model of the motor primitive has yet considered effort minimization. Effort minimization is a widely accepted framework in motor control because the standard deviation of muscle activity is proportional to the muscle activity itself, which allows effort minimization to effectively minimize motor noise (Jones et al., 2002) or endpoint variance (Harris and Wolpert, 1998). Thus, it is natural to introduce effort minimization into conventional frameworks of motor primitives. Here, I analytically and numerically demonstrate that a motor primitive framework can reproduce context-dependent decay with the assumptions of both error and effort minimization.

## 2. Materials and methods

### 2.1. General framework

**Figure 1:**
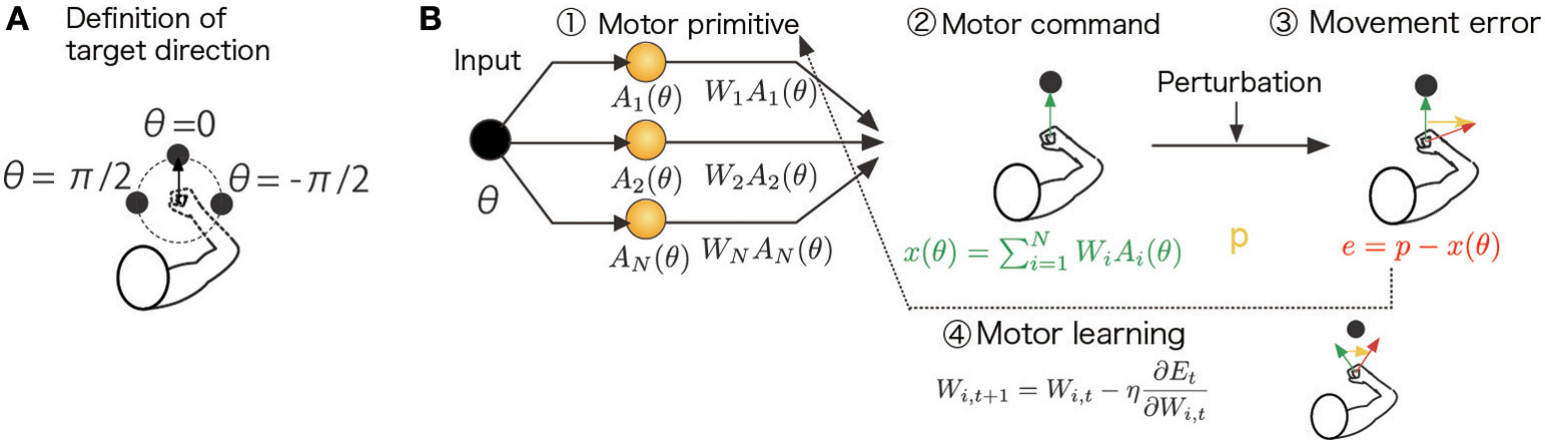
**Schematic diagram of the computational model and assumed task**. **(A)** Definition of the target direction. θ = 0 is defined as the target for the straight-forward reaching movement. **(B)** The target direction (desired movement direction) θ_*t*_ determined the activities of the motor primitives *A_i_*(θ_*t*_) in the *t*-th trial. The task was to generate a motor command *x_t_* to compensate for an environmental change (perturbation) *p_t_*. The motor command *x_t_* was determined by a linear combination of motor primitive activities, $\sum_{i=1}^{N}W_{i}A_{i}(\theta_{t})$, and these linear coefficients were modified to minimize a cost function *E_t_* (e.g., the squared movement error and effort): $W_{i,t+1}=W_{i,t}-\eta \frac{\partial E_{t}}{\partial W_{i,t}}$. These procedures are summarized in the Summary of the Motor Primitive Framework Section in the Materials and Methods.

The present study focused on unimanual reaching movements in the horizontal plane toward radially distributed targets whose directions were defined as θ ∈ [−π, π] (Figure 1A). During each reaching movement, an unpredictable perturbation *p*, such as a force field (Shadmehr and Mussa-Ivaldi, 1994) or a visuomotor transformation (Krakauer et al., 1999), was imposed and yielded a movement error *e* (Figure 1B). The aim of the task was to accurately reach to a given target by generating an additional motor command *x* to compensate for the movement error, i.e., *e* = *p* − *x*. Here, following several previous studies, I assumed that *x* represents the lateral force in the force adaptation (Yokoi et al., 2011; Brayanov et al., 2012; Yokoi et al., 2014) or the movement angle in the visuomotor rotation (Tanaka et al., 2009; Taylor et al., 2012) at the time of the peak velocity because these values are considered to represent the degree of adaptation in a feedforward controller, which many motor learning studies have focused on. That is, *p* represents an applied lateral force in a curl force field paradigm (the units of *p*, *x*, and *e* are newtons) and a rotated movement angle in a visuomotor rotation paradigm (the units of *p*, *x*, and *e* are degrees). Notably, the movement error does not include the target direction. The motor primitive framework assumes that the motor commands of the baseline trials (before experiencing the adaptation paradigm) *x_b_* can be appropriately generated, i.e., *x_b_* = τ_θ_ in force field adaptation, where τ_θ_ is the desired torque to achieve the reaching movements toward θ, and *x_b_* = θ in the visuomotor rotation adaptation. When the perturbation *p* is applied and the compensatory motor command *x* is generated, the baseline motor command is distorted as *x_b_* − *p* + *x*, resulting in movement errors that can be written as *e* = τ_θ_ − (*x_b_* − *p* + *x*) = *p* − *x* in the force field adaptation and *e* = θ − (*x_b_* − *p* + *x*) = *p* − *x* in the visuomotor rotation adaptation.

Following previous motor primitive models (Thoroughman and Shadmehr, 2000; Donchin et al., 2003; Tanaka et al., 2009; Yokoi et al., 2011; Brayanov et al., 2012; Taylor et al., 2012; Yokoi et al., 2014), we assumed that the recruitment pattern of the motor primitives or the motor primitive activities are determined by a target direction θ and the Gaussian

(1) $$A_{i}(\theta )=\exp\left(-\frac{1}{2\sigma_{i}^{2}}{\left\|\theta -\varphi_{i}\right\|}^{2}\right),$$

where the scaling parameter σ_*i*_ = σ is independent of *i*; φ_*i*_ is the preferred direction (PD) of the *i*-th primitive, which is randomly sampled from a uniform distribution in the range [−π, π]; ||θ|| is a periodic function ||θ|| = ||θ + 2π|| such that ||θ|| = θ for −π ≤ θ < π (θ = 0 is defined as the target for a straight-forward reaching movement, Figure 1A), and *i* = 1, …, *N*, and *N* is the number of primitives. The scaling parameter σ controls the number of primitives responsible for a reaching movement toward θ because the *i*-th primitive is activated independent of θ when σ is sufficiently large (i.e., the number of recruited primitives is large) and because the primitive shows its activity only in the case θ = φ_*i*_ when σ is sufficiently small (i.e., the number of recruited primitives is small). The PD determines the reaching direction in which the *i*-th primitive shows its maximal activity. Because the PD was randomly sampled in each simulation run, the simulation results were variable across runs. The standard deviation of the simulation results is plotted in Figures 3D–I.

The compensatory motor command for the reaching movement toward θ, *x*(θ), is a linear summation of the motor primitive activities *A*_1_(θ), …, *A_N_*(θ):

(2) $$x(\theta )=\sum_{i=1}^{N}W_{i}A_{i}\left(\theta \right),$$

where the adaptable weight value *W_i_* determines how the *i*-th primitive contributes to the generation of the motor command. Each weight *W_i_* is modified by

(3) $$W_{i,t+1}=W_{i,t}-\eta \frac{\partial E_{t}}{\partial W_{i,t}}$$

(gradient descent rule) at the *t*-th trial to reduce the cost function *E_t_*, where the positive constant η > 0 denotes the learning rate. Conventional motor primitive frameworks assume that *E_t_* consists of the squared movement error *e*^2^_*t*_ and the sum of squared weight values $\sum_{i=1}^{N}W_{i,t}^{2}$, which leads to the learning rule of Equation (12) in the Results section or to context-independent decay of motor memory. Here, I suppose that the cost function consists of the squared movement error and the squared motor command amplitude (effort) *x*^2^_t_, which leads to the learning rule of Equation (15) in the Results section or to context-dependent decay of motor memory. When $E_{t}=\frac{1}{2}e_{t}^{2}+\frac{\lambda_{1}}{2}\sum_{i=1}^{N}W_{i,t}^{2}+\frac{\lambda_{2}}{2}x_{t}^{2}$, Equation (3) can be written as

(4) $$W_{i,t+1}=\left(1-\eta \lambda_{1}\right)W_{i,t}-\eta \lambda_{2}x_{t}\left(\theta_{t}\right)A_{i}\left(\theta_{t}\right)+\eta e_{t}A_{i}\left(\theta_{t}\right),$$

where θ_*1*_ and θ_*2*_ are regularization parameters to determine the tradeoff between the minimization of the squared movement error, the minimization of the sum of the squared weight values, and the minimization of effort.

### 2.2. Summary of the motor primitive framework

The motor primitive framework can be summarized as follows. Setting the parameters σ, η, θ_*1*_, θ_*2*_, *p* to certain values, *e*_0_ = 0, *W*_0_ = 0, φ_*i*_ to a randomly sampled value in the range [−π, π], and the target direction θ_*t*_ ∈ [−π, π] to a certain value at the *t*-th trial yields the following:

(5) $$\begin{array}{l} \left(\text{Determining recruitment pattern of motor primitives}\right)\\ \;A_{i}(\theta_{t})=\exp\left(-\frac{1}{2\sigma^{2}}{\left\|\theta_{t}-\varphi_{i}\right\|}^{2}\right). \end{array}$$

(6) $$(\text{Generation of a motor command})\;x_{t}\left(\theta_{t}\right)\text{ ​}=\text{​​}\sum_{i=1}^{N}W_{i,t}A_{i}\left(\theta_{t}\right).$$

(7) $$(\text{Observation of a movement error})\;e_{t}=p_{t}-x_{t}\left(\theta_{t}\right).$$

(8) $$\begin{array}{l} \left(\text{Update of linear coefficients}\right)\\ W_{i,t+1}=\left(1-\eta \lambda_{1}\right)W_{i,t}-\eta \lambda_{2}x_{t}\left(\theta_{t}\right)A_{i}\left(\theta_{t}\right)+\eta e_{t}A_{i}\left(\theta_{t}\right). \end{array}$$

Notably, with the assumptions of the motor primitive frameworks and the gradient descent as the learning rule, these procedures are exact and without approximation. This motor primitive framework can explain trial-dependent changes in the movement error and generalization (Thoroughman and Shadmehr, 2000; Donchin et al., 2003).

### 2.3. Generalization function

Learning effects trained with reaching movements toward θ_*t*_ were generalized to untrained reaching movements toward θ through a generalization function *G*(θ_*t*_, θ), where θ_*t*_ and θ were assumed to be the target directions in the training and test trials, respectively. Test trials indicate the trials to probe learning effects during or after the training trials. Equation (4) indicates the learning process during the training of reaching movements toward θ_*t*_. The degree of generalization from the reaching movements toward θ_*t*_ to other reaching movements toward θ can be derived by multiplying *A_i_*(θ) and summing across all values of *i* in Equation (4):

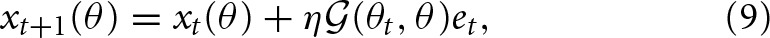



where the generalization function is defined as 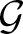
$(\theta_{t},\theta )=\sum_{i=1}^{N}A_{i}(\theta_{t})A_{i}(\theta )$ and both λ_1_ and λ_2_ are set to 0 for simplicity (cases in which λ_1_ ≠ 0 or λ_2_ ≠ 0 are discussed in the Results section). The generalization function *G*(θ_*t*_, θ) is defined as the inner product of the recruitment pattern of motor primitives during reaching toward θ_*t*_ and those for reaching toward θ. Larger overlaps between the two recruitment patterns result in greater generalization. In addition, the generalization function is dependent only on σ (see Equation (10) described below) and is independent of other parameters, including the perturbation *p*.

More intuitively, when the number of primitives *N* is sufficiently large and η is divided by *N* (most conventional motor primitive models assume these conditions), the generalization function can be approximated as

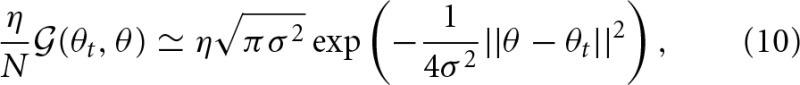



which indicates that the closer the target direction in the test trials (θ) is to the direction in the training trials (θ_*t*_), the greater the generalization. In addition, the generalization function depends only on σ. Although this derivation of Equation (10) requires a sufficiently large *N*, *N* does not significantly influence the shape of *G*(θ_*t*_, θ) (Takiyama and Okada, 2012a). Thus, Equation (10) permits an intuitive understanding of the results of the current study. Furthermore, this type of generalization function suitably explains the actual data from a wide range of motor learning experiments (Thoroughman and Shadmehr, 2000; Donchin et al., 2003; Tanaka et al., 2009; Yokoi et al., 2011; Brayanov et al., 2012; Taylor et al., 2012; Yokoi et al., 2014).

### 2.4. State space modeling

Although the motor primitive framework assumed trial-by-trial variations in the weight value, *W*, it was possible to model only the trial-by-trial variation of the motor command *x*. In conventional state space models, motor learning is modeled as an optimization process regarding motor commands:

(11) $$x_{t+1}=x_{t}-\eta \frac{\partial E_{t}}{\partial x_{t}},$$

where *E_t_* is a cost function at the *t*-th trial, e.g., the squared movement error. Notably, Equation (0) is equivalent to Equation (11) when θ = θ_*t*_ and $\sqrt{\pi \sigma^{2}}\eta$ is redefined as η. Thus, this state space model can be viewed as a motor learning model that focuses only on the trial-by-trial variation of the motor commands without assuming any generalization function. A previous study demonstrated that the state space model fits the data well if context-dependent decay and the generalization function are heuristically introduced into this model (Ingram et al., 2013), i.e., *x*_*t*+1_(θ) = (1 − ηλ_2_
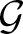
_1_(θ_*t*_, θ))*x_t_*(θ) + η
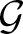
_2_(θ_*t*_, θ) *e_t_* (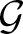
_1_ and 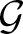
_2_ are some functions).

### 2.5. Numerical simulation

**Figure 2:**
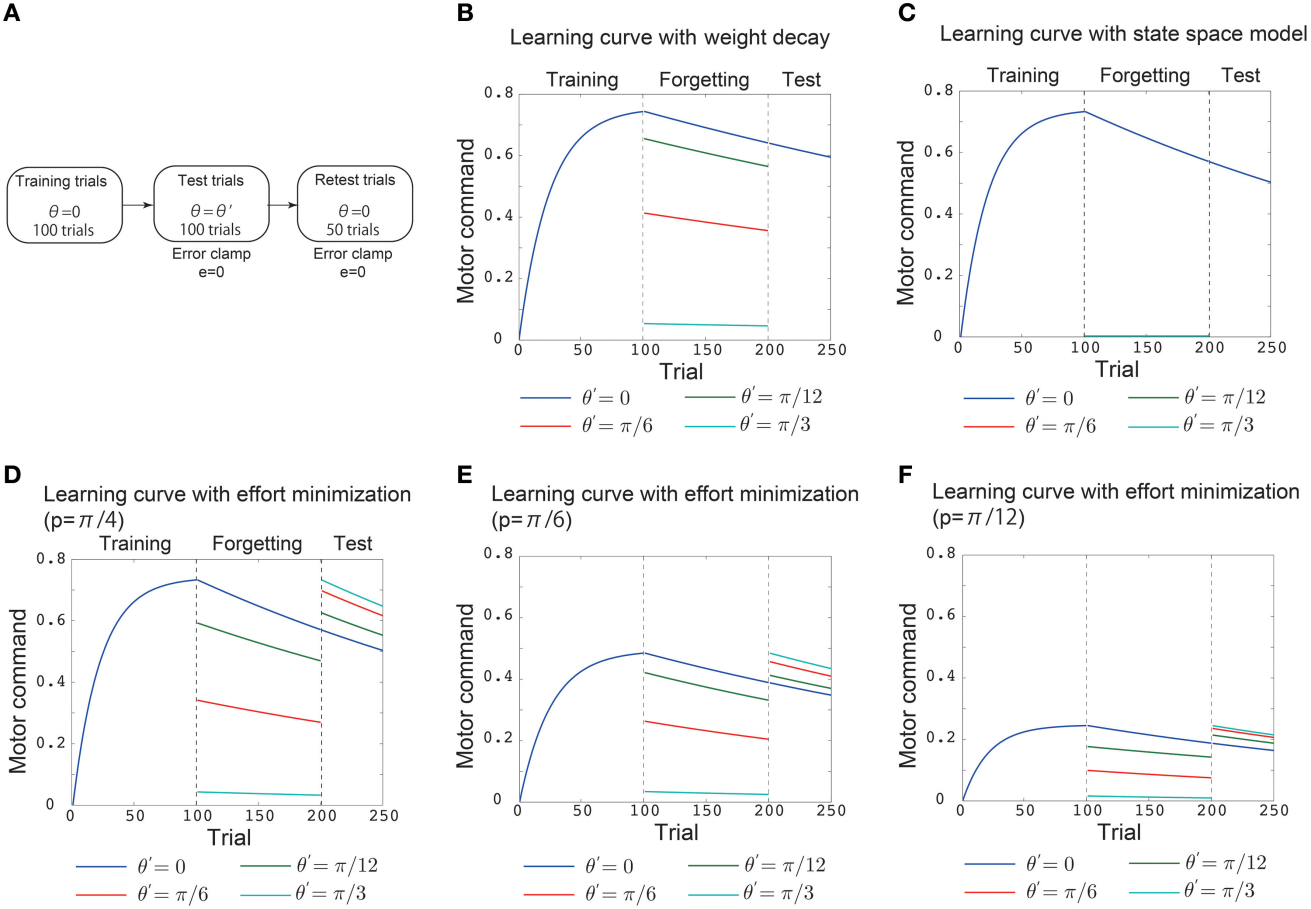
**Results of simulation 1**. **(A)** Simulated experimental setting in simulation 1. This setting consisted of 100 training trials, 100 test trials, and 50 retest trials. In the training trials, the (simulated) subjects were required to adapt to *p* = π/4 during reaching movements toward θ = 0. In the test trials, “error clamp” trials in which the movement error *e* was forcibly set to 0 were imposed. In these trials, θ = 0 (blue line in **B–F**), θ = π/12 (green line in **B–F**), θ = π/6 (red line in **B–F**), and θ = π/4 (cyan line in **B–F**). After these test trials, another 50 error clamp trials were imposed with θ = 0. The vertical black dotted lines denote the trials in which the training trials were switched to the test trials and the test trials were switched to the retest trials. **(B)** Trial-by-trial variation in the motor command *x* in the motor primitive framework with a weight decay. **(C)** Trial-by-trial variation in the motor command in the state space model with effort minimization. **(D)** Trial-by-trial variation in the motor command in the motor primitive framework with effort minimization. **(E,F)** Trial-by-trial variation in the motor command in the motor primitive framework with effort minimization when *p* = π/6 in **(E)** and *p* = π/12 in **(F)**.

**Figure 3:**
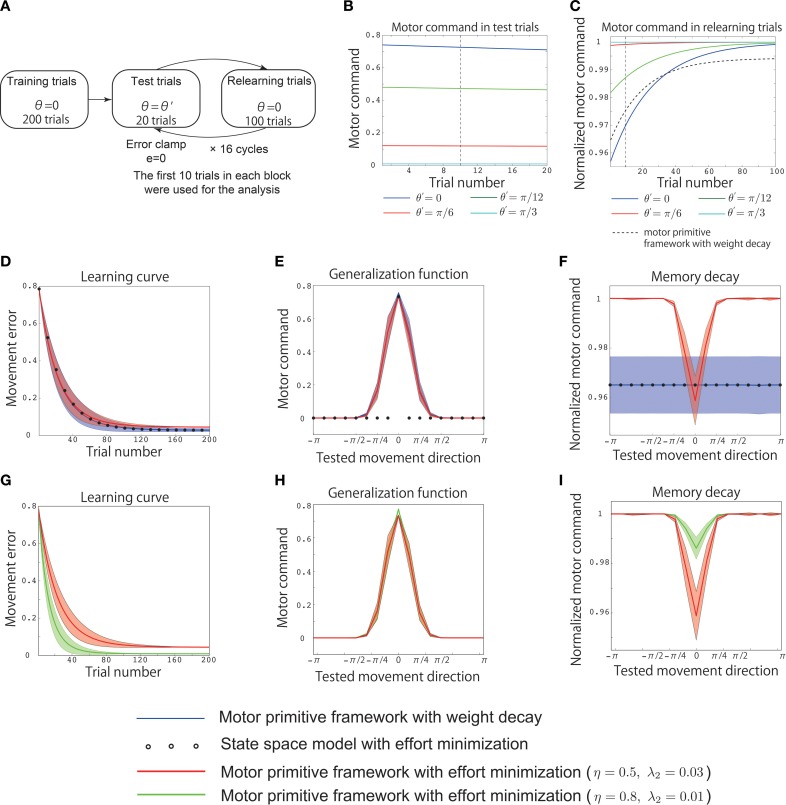
**Results of simulation 2**. **(A)** Simulated experimental setting for simulation 2. Training trials were conducted for 200 trials with θ = 0. After the training, 20 test trials with $\theta^{\prime }=-\pi +2\pi \frac{k}{K}$ and 100 relearning trials with θ = 0 were alternately simulated for *K* cycles, where *K* = 16, and the integer *k* was pseudorandomly sampled from the range [0, *K* − 1] to assume different values in each cycle. In the test trials, the movement error was forcibly set to 0 assuming error clamp trials. **(B)** Trial-by-trial variation of the motor command *x* in the test trials with the motor primitive framework with effort minimization, θ′ = 0 (blue line), θ′ = π/12 (green line), θ′ = π/6 (red line), and θ′ = π/3 (cyan line). The vertical black dotted line indicates the 10th test trial. **(C)** Trial-by-trial variation of the motor command in the relearning trials with the motor primitive framework with effort minimization. The black dotted line denotes the trial-by-trial variation of the motor command in the relearning trials with the motor primitive framework with weight decay (independent of θ′). The vertical black dotted line indicates the 10th relearning trial. **(D)** Learning curves. The horizontal axis indicates the trial number, and the vertical axis denotes the movement error *e_t_*. The red line, blue line, and circle denote the trial-by-trial variation of *e_t_* averaged across 20 simulation runs in the motor primitive framework with effort minimization, the framework with weight decay, and the state space model with effort minimization, respectively. The red and blue shaded areas indicate the standard deviations of the learning curves in the 20 simulation runs in the motor primitive framework with effort minimization and those with weight decay, respectively. After 200 trials, the motor commands *x*_200_(0) converged to *x*_0_ in all three models. **(E)** Generalization function averaged across 20 simulation runs. The horizontal axis indicates the tested movement direction θ′, and the vertical axis indicates the motor command *x*(θ′). To draw the generalization function, I averaged the motor commands of the initial 10 test trials in each cycle (the 10th test trial is indicated by the vertical black dotted line in **B**). The red and blue shaded areas indicate the standard deviation of the generalization function in the 20 simulation runs in the motor primitive frameworks with effort minimization and weight decay, respectively. **(F)** Context-dependent decay investigated in the relearning phase with θ′ = 0. The horizontal axis indicates the tested movement direction θ′, and the vertical axis indicates the normalized motor command in relearning trials. The normalized motor command was defined as $\frac{1}{10}\sum_{t=1}^{10}x_{\text{relearning},\text{t}}(0)/x_{0}$, where *x*_relearning, t_(0) is a motor command at the *t*-th relearning trial in each cycle, i.e., I averaged the motor commands of the initial 10 relearning trials in each cycle (the 10th relearning trial is indicated by the vertical black dotted line in **C**) and divided the averaged value by *x*_0_. The red and blue shaded areas indicate the standard deviations of the memory decays in the 20 simulation runs in the motor primitive frameworks with effort minimization and weight decay, respectively. **(D,E,F)** Parameter sensitivities of the learning curve, the generalization function, and the context-dependent memory decay in the motor primitive with effort minimization. The red lines denote the generalization function and the context-dependent decay with η = 0.5 and λ = 0.03, and the green lines denote those with η = 0.8 and λ = 0.01. The red and green shaded areas indicate the standard deviations of the learning curves in the 20 simulation runs in the motor primitive frameworks with effort minimization when η = 0.5 and λ = 0.03 and η = 0.8 and λ = 0.01, respectively.

The learning rates in the motor primitive framework and state space modeling, i.e., η in Equations (12) and (15) and η in Equation (14), were set to 0.5 and 0.04, respectively. The rates of forgetting in weight decay (λ_1_ in Equation 14) and in state space modeling (λ_2_ in Equation 14) were set to 0.0015/η. The rate of forgetting in effort minimization in a motor primitive framework (λ_2_ in Equation 15) was set to 0.03/η. The number of motor primitives *N* was set to 100, and the perturbation *p* was set to π/4. The three types of models considered in the current study, i.e., the motor primitive framework with weight decay, the state space model with effort minimization, and the motor primitive with effort minimization, produced nearly identical learning curves using these parameters (Figure 3D). To investigate the parameter dependencies, *p* was set to π/6 or π/12 in Figures 2E,F, respectively, and η and λ_2_ were set to 0.8 and 0.01 in Figures 3H,I.

## 3. Results

I analytically and numerically investigated a condition in which the decay of motor memory and the availability of learning effects were context dependent. The present study assumed a task involving unimanual reaching movements toward radially distributed target directions in the horizontal plane (Figure 1A; detailed descriptions of the following framework, analytical calculations, and each parameter value were provided in the Materials and Methods section). The goal of the task was to reach to the target accurately in a situation in which executed movements were perturbed by changes in the environment *p*, e.g., the external force generated by a manipulandum (Shadmehr and Mussa-Ivaldi, 1994) or visuomotor transformation (Krakauer et al., 1999). The motor command *x* to compensate for a perturbation was modeled by the summation of the activities of the motor primitives as $x(\theta )=\sum_{i=1}^{N}W_{i}A_{i}(\theta )$, where the adaptable *W_i_* represents how the *i*-th primitive contributes to the production of the motor command and *A_i_*(θ) is the activity of the *i*-th primitive determined by the target direction. The current study assumed that the recruitment pattern of motor primitives was determined by the target direction. The movement error in the *t*-th trial can thus be expressed as *e_t_* = *p_t_* − *x_t_*. To minimize the squared movement error, the squared sum of the weight values, and the squared motor command amplitude (effort), *W_i_* was modified in each trial (Equation 4).

### 3.1. Weight decay

Conventional models of motor primitives assume weight decay (Thoroughman and Shadmehr, 2000; Donchin et al., 2003; Tanaka et al., 2009; Yokoi et al., 2011; Brayanov et al., 2012; Hirashima and Nozaki, 2012; Takiyama and Okada, 2012a; Taylor et al., 2012; Yokoi et al., 2014), i.e., the cost function *E_t_* to be minimized is defined as a weighted sum of the squared error and the squared sum of weight values $E_{t}=\frac{1}{2}e_{t}^{2}+\frac{\lambda_{1}}{2}\sum_{i=1}^{N}W_{i,t}^{2}$, where a regularization parameter λ_1_ determines the trade-off between error minimization and weight value minimization. The learning rule can thus be written as

(12) $$W_{i,t+1}=\left(1-\eta \lambda_{1}\right)W_{i,t}+\eta e_{t}A_{i}\left(\theta_{t}\right),$$

where λ_1_ indicates the forgetting rate and η denotes the learning rate: The larger λ_1_ is, the faster the motor memory decays, and the larger η is, the faster the movement error is minimized. In addition, the larger the *i*-th primitive is activated, the faster the motor memory can be embedded in the *i*-th primitive because the learning rate η is modulated by *A_i_* in Equation (12). However, the forgetting rate is not modulated by *A_i_*, which results in motor memory decays in the trial when the *i*-th primitive is not activated. The assumed situation in this equation was an adaptation process in which the subjects adapted to a perturbation with reaching movements toward θ_*t*_. Equation (12) can be rewritten as a recursive equation of *x*: 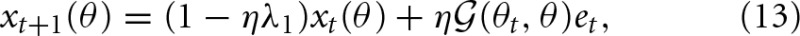 where 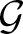
$(\theta_{t},\theta )=\sum_{i=1}^{N}A_{i}(\theta_{t})A_{i}(\theta )$ is the generalization function (detailed derivation and description are provided in the Generalization Function section in the Materials and Methods). This equation indicates how the learning effects trained during reaching toward θ_*t*_ are generalized to the untrained reaching movements toward θ in each trial. The generalization function indicates that the learning effects can be largely observed in test trials when tested movement direction θ is close to the trained movement direction θ_*t*_. Although this generalization function can be fit well to a wide range of experimental data (Thoroughman and Shadmehr, 2000; Donchin et al., 2003; Tanaka et al., 2009; Yokoi et al., 2011; Brayanov et al., 2012; Taylor et al., 2012; Yokoi et al., 2014), the decay of motor memory, the first term in the right side of Equation (13), is independent of the target directions θ and θ_*t*_ (i.e., the context-independent memory decay), in contrast to the results of a previous behavioral experiment (Ingram et al., 2013). Thus, the motor primitive framework with weight decay does not reproduce context-dependent memory decay.

### 3.2. Effort minimization in a state-space model

In addition to the motor primitive framework, the state space model is another widely used model of motor learning (Scheidt et al., 2001; Smith et al., 2006; Lee and Schweighofer, 2009). The state space model focuses on the trial-by-trial variation of the motor command *x* rather than the trial-by-trial variation of the weight value *W*. Thus, weight decay cannot be assumed in the state space model because this model does not assume any weight value. Memory decay was modeled as effort minimization in the state space model using the cost function $E_{t}=\frac{1}{2}e_{t}^{2}+\frac{\lambda_{2}}{2}x_{t}^{2}$, i.e., a weighted sum of squared error and effort. Optimizing this cost function led to the minimization of movement error and effort. The learning rule can be written as

(14) $$x_{t+1}\left(\theta_{t}\right)=\left(1-\eta \lambda_{2}\right)x_{t}\left(\theta_{t}\right)+\eta e_{t},$$

where the memory decay and the availability of learning effects are context independent, also in contrast to the results of previous behavioral experiments (Thoroughman and Shadmehr, 2000; Donchin et al., 2003; Ingram et al., 2013). Although the state space model can be fit well to actual data by heuristically introducing context-dependent memory decay and a generalization function (Ingram et al., 2011, 2013), such modeling leaves the question of what is optimized in motor learning. While more complicated versions of state space models can be considered (Smith et al., 2006; Lee and Schweighofer, 2009; Takiyama et al., 2009; Takiyama and Okada, 2011), context-dependent memory decay cannot be reproduced. Thus, the state space model is a powerful model in some cases (Scheidt et al., 2001; Smith et al., 2006; Lee and Schweighofer, 2009), but is not suitable for modeling and interpreting the context-dependent decay of motor memory and generalization in an optimization framework.

### 3.3. Effort minimization in the motor primitive framework

In the previous two models, the parameter being optimized differed: the weight value was minimized in the conventional motor primitive framework, while effort was minimized in the state space model. Although the functional roles of weight decay in motor learning remain unclear (cf. Hirashima and Nozaki, 2012), effort minimization has been reported to be effective in minimizing motor noise (Jones et al., 2002) and endpoint variance (Harris and Wolpert, 1998). Furthermore, some previous studies have reported that not only movement error but also effort is minimized in the adaptation to a force field (Emken et al., 2007; Huang et al., 2012) and a visuomotor transformation (Huang and Ahmed, 2014). Thus, I propose a motor primitive framework with effort minimization. When *E_t_* is defined as $E_{t}=\frac{1}{2}e_{t}^{2}+\frac{\lambda_{2}}{2}x_{t}^{2}$, motor learning can be modeled as

(15) $$W_{i,t+1}=W_{i,t}-\eta \lambda_{2}x\left(\theta_{t}\right)A_{i}\left(\theta_{t}\right)+\eta e_{t}A_{i}\left(\theta_{t}\right).$$

The second term in the right side of Equation (15) is a memory decay that can be modulated by *x*(θ_*t*_) and *A_i_*(θ_*t*_); the larger the *i*-th primitive is activated, the faster the motor memory embedded in the primitive decays. Equation (15) yields a recursive equation for motor commands:

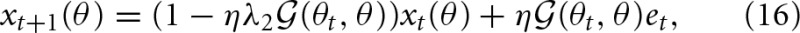



where both memory decay and error minimization are context dependent. When trained with reaching movement toward θ_*t*_ and tested with movements toward θ, the motor memory decays faster in the test trials when the tested movement direction is close to the trained movement direction because the forgetting rate ηλ_2_
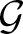
(θ_*t*_, θ) is maximal when θ = θ_*t*_. This result is consistent with results of a previous experiment (Ingram et al., 2013). Taken together, effort minimization in a motor primitive framework is the only candidate that can simultaneously reproduce context-dependent memory decay and generalization.

### 3.4. Simulation 1: validation of the analytical consideration

To validate the analytical considerations, I conducted numerical simulations (Figures 2, 3). The first simulation consisted of 100 training trials, 100 test trials, and 50 retest trials (Figure 2A). In the training trials, the (simulated) subjects were required to adapt to *p* = π/4 in reaching movements toward θ = 0. The test trials were “error clamp” trials in which the movement error *e* was forcibly set to 0 to enable a clear discussion of learning effects by excluding any adaptation process (Scheidt et al., 2000). These test trials were simulated to investigate how the learning effects trained in training trials are generalized to reaching movements toward θ = 0 (blue line), θ = π/12 (green line), θ = π/6 (red line), or θ = π/4 (cyan line). Notably, the error clamp trials also enabled a clear discussion of forgetting processes because no adaptation can be assumed to occur during the trials. Thus, the memory of learning effects trained in the training trials decayed during the test trials depending on θ. To investigate how the target direction (context) affected the decay of the motor memory, another 50 error clamp trials were imposed with θ = 0 after the test trials (Figure 2A).

The above simulated experimental setting enabled an investigation of whether the decay of motor memory and the availability of learning effects are context dependent or context independent. If the availability of learning effects is context dependent, the learning effects trained with θ = 0 in the training trials will be generalized to other reaching movements in the test trials, and the motor command toward the other target directions *x*(π/12), *x*(π/6), and *x*(π/4) will not be 0 in those trials. By contrast, if the availability of learning effects is context independent, then no generalization should be observed, and *x*(π/12), *x*(π/6), and *x*(π/4) are 0 in the test trials. If the memory decay is context independent, the motor commands in the retest trials should be independent of the target direction in the test trials, i.e., the blue, green, red, and cyan lines that indicate *x*(0) should converge to the same value in the retest trials. This result would indicate that the motor memory decays at the same rate in the test trials independent of the target direction. However, if the memory decay is context dependent, then the motor commands in the retest trials should depend on the target direction in the test trials, and the blue, green, red, and cyan lines that indicate *x*(0) should diverge to different values in the retest trials. This result would indicate that the motor memory decays with different rates in the test trials depending on the target direction.

In the motor primitive framework with weight decay Equation (12), the availability of learning effects is context dependent (Figure 2B, the blue, green, red, cyan lines in the test trials). However, the memory decay is context independent (Figure 2B, all the lines converged to the same value in retest trials). In the state space model with effort minimization Equation (14), neither the availability of learning effects nor the memory decay is context dependent (Figure 2C). Thus, these conventional frameworks are not sufficient to reproduce both generalization and context-dependent memory decay.

By contrast, the motor primitive framework with effort minimization Equation (15) reproduced both generalization and the context-dependent memory decay (Figure 2D). Furthermore, these generalization and context-dependent decay were independent of the magnitude of perturbation (Figures 2E,F). Thus, my analytical considerations were validated; context-dependent memory decay is a result of effort minimization in the motor primitive framework.

### 3.5. Simulation 2: reproduction of previous experimental data

Next, I reproduced the results of a previous behavioral experiment (Ingram et al., 2013) using the assumptions of a similar experimental setting (Figure 3). The training involved 200 trials with θ = 0. After these trials, the motor command *x*_200_(0) converged to *x*_0_ (Figure 3D). After the training, 20 test trials with $\theta^{\prime }=-\pi +2\pi \frac{k}{K}$ and 100 relearning trials with θ = 0 were alternately simulated for *K* cycles in which the integer *k* was peudorandomly sampled from the range [0, *K* − 1] to take different value in each cycle and *K* was 16. Once the integer *k* was sampled, *k*, or θ′ was fixed across the 20 test trials. The movement error was forcibly set to 0 in the test trials (error clamp trials). These test trials enabled to investigate the generalization. If the availability of the learning effects is context independent, motor commands in test trials will be 0 except for the case when θ′ = 0. By contrast, the availability of the learning effects is context dependent, the learning effects trained in training or relearning trials will be generalized to other reaching movements in test trials. Because the magnitude of the motor commands (motor memory) decayed during the test trials (Figure 3B), I averaged the motor commands in the initial 10 test trials to obtain the generalization function shown in Figures 3E,H to eliminate the effects of decay on the function.

After the test trials, 100 relearning trials with θ = 0 were imposed to investigate the context dependence of the memory decay. If the decay rate of the memory was independent of θ′ in the test trials, the motor commands in the relearning trials should have the same values independent of θ′. By contrast, if the decay rate of the memory depended on θ′, the motor commands in the relearning trials should have different values depending on θ′. Because the motor commands converged to the same value during the relearning trials (Figure 3C), I averaged the motor commands in the initial 10 relearning trials to obtain the context-dependent memory decay in Figures 3F,I to eliminate the effects of relearning on the function of the context-dependent memory decay. In addition, based on a previous study (Ingram et al., 2013), the motor commands in relearning trials were denoted after normalizing by *x*_200_(0) in the training trials (Figures 3C,F,I).

Similar to the previous section, Simulation 1: Validation of the Analytical Consideration, neither the motor primitive framework with weight decay nor the state space model with effort minimization reproduced the context-dependent memory decay in an experimental setting similar to that of the previous study (Ingram et al., 2013) (blue line and circles in Figures 3E,F). By contrast, the motor primitive framework with effort minimization reproduced both the context-dependent memory decay and the generalization in this experimental setting (red line in Figures 3E,F). Furthermore, these results were not sensitive to parameter changes (Figures 3G–I); although the magnitude of the memory decay was reduced when λ_2_ was small, the context dependencies of the memory decay and the generalization were invariant. Thus, this framework was able to reproduce the generalization and context-dependent decay within an experimental setting similar to that of the previous study (Ingram et al., 2013). Taken together, the context-dependent decay and the generalization were the results of optimizing the effort and movement error in the motor primitive framework.

## 4. Discussion

In the current study, I revealed that context-dependent decay is evidence of effort minimization in motor learning by extending a motor primitive framework. The conventional motor primitive framework succeeded in reproducing the availability of the learning effects trained with reaching movements toward θ in other reaching movements (Thoroughman and Shadmehr, 2000; Donchin et al., 2003; Tanaka et al., 2009; Yokoi et al., 2011; Brayanov et al., 2012; Taylor et al., 2012; Yokoi et al., 2014). Although the conventional models of motor learning support the existence of memory decay in motor learning processes (Thoroughman and Shadmehr, 2000; Scheidt et al., 2001; Donchin et al., 2003; Smith et al., 2006; Tanaka et al., 2009; Yokoi et al., 2011; Brayanov et al., 2012; Hirashima and Nozaki, 2012; Takiyama and Okada, 2012a; Taylor et al., 2012; Yokoi et al., 2014), these conventional models assume that the rate at which memory decays is independent of the target direction or assume context-independent decay of motor memory. Context-independent decay is equivalent to the minimization of weight values (weight decay) in a motor primitive framework Equations (12) and (13) and effort minimization in a state space model Equation (14). Nevertheless, a recent behavioral experiment (Ingram et al., 2013) reported that the rate of memory decay is context dependent. Although motor learning has been modeled by an optimization framework, context-dependent decay raises the following question: what is optimized in motor learning? Here, based on the results of recent behavioral experiments (Emken et al., 2007; Huang et al., 2012; Huang and Ahmed, 2014), I introduced effort minimization into a motor primitive framework and concluded that context-dependent decay results from effort minimization in the motor primitive framework Equations (15) and (16), Figures 2D, 3E,F). Furthermore, this context dependence of the motor memory decay was not sensitive to the parameters η, λ_2_, and *p* (Figures 2E,F, 3G–I). This finding permits the seamless connection of conventional models of motor learning with the recent finding of context-dependent decay.

The ability of effort minimization to reproduce context-dependent decay is related to activity-dependent memory decay. In the error clamp trials, Equation (15) can be written as *W*_*i*, *t*+1_ = *W*_*i*, *t*_ − ηλ_2_*x_t_A_i_*(θ_*t*_), which indicates that the more the *i*-th primitive is activated in the *t*-th trial, the faster the memory embedded in the primitive decays. In other words, if the *i*-th primitive is not activated in a trial, the memory embedded in the primitive can bE completely maintained during the error clamp trials. When trained with θ = 0 and test trials imposed with θ = π/4 (cyan line in Figures 2D–F), the recruitment patterns differed greatly between the training and test trials, and thus the motor memories embedded in the motor primitives that were activated when θ = 0 were maintained in the test trials with θ = π/4. Thus, after the test trials with θ = π/4, the motor memory was maintained, and the magnitudes of the motor commands in the retest trials were not significantly different from the motor commands in the latter half of the training trials (Figures 2D–F). When trained with θ = 0 and test trials imposed with θ = 0 (blue line in Figures 2D–F), the same primitives were activated during the training and test trials, which led to faster forgetting of the motor memory in the test trials. Thus, after the test trials with θ = 0, the motor memory decays and the magnitudes of the motor commands in the retest trials were smaller than the motor commands in the latter half of the training trials (Figures 2D–F). By contrast, weight decay entails activity-independent memory decay. In the error clamp trials, Equation (12) can be written as *W*_*i*, *t*+1_ = (1 − λ_1_)*W*_*i*,*t*_, which indicates that the motor memory embedded in the *i*-th primitive decays trial-by-trial independent of the activity of the *i*-th primitive or the target direction in the test trials (Figure 2B). Hence, activity-dependent memory decay is responsible for the association of effort minimization with the context-dependent decay of motor memory.

Functional roles of effort minimization in motor learning needs to be discussed. One possible functional role of effort minimization is to reduce mean squared movement error when a motor command includes signal-dependent noise (the noise whose standard deviation is proportional to its mean value), i.e., *y* = *x* + ξ, where *y* is a motor command that includes motor noise, *x* is a noiseless motor command, and ξ is a motor noise with a mean of 0 and a variance of λ_2_x^2^. In this case, the mean squared error can be written as follows: 〈*e*^2^_*t*_〉 = (*p_t_* − 〈 *y_t_*〉)^2^ + 〈(*y_t_* − 〈*y_t_*〉)^2^〉 = *e*^2^_*t*_ + λ_2_*x*^2^_*t*_, where 〈*f*(ξ_*t*_)〉 indicates *f*(ξ_*t*_) averaged across all possible realizations 〈*f*(ξ_*t*_)〉 = ∫ *d*ξ_*t*_*p*(ξ_*t*_)*f*(ξ_*t*_). This equation indicates that the mean squared movement error consists of constant error, (*p_t_* − *x_t_*)^2^ and variable error 〈(*y_t_* − *x_t_*)^2^〉 = λ_2_*x*^2^_*t*_ (Schmidt and Lee, 1988). The constant error represents the bias of the motor command. The smaller (larger) the motor command is down-regulated (up-regulated), e.g., *y_t_* = *p_t_* − *b* + ξ_*t*_ (*y_t_* = *p_t_* + *b* + ξ_*t*_), where *b* is bias, the larger the constant error. By contrast, the variable error represents how variable the motor command is in each trial. The more variable the motor command, the larger the variable error. When the motor noise is signal dependent, effort minimization can decrease the trial-by-trial variability such that effort minimization decreases the variable error and the mean squared movement error.

One limitation of this study must be discussed. Although a previous behavioral experiment reported that both context-dependent decay and the generalization function can be modeled by Gaussian functions, the width of the function was wider for context-dependent decay than for the generalization function (Ingram et al., 2013). In Equation (16), the width of the Gaussian was the same for the two functions; the reason for the difference in the widths reported in the previous study remains unclear. A possible solution is the introduction of a multi-rate concept (Smith et al., 2006) into the motor primitive framework (Tanaka et al., 2012). In this concept, the motor memory consists of at least two processes, i.e., fast and slow processes in which, roughly speaking, the fast processes, including a large forgetting and a large learning rate, are involved when the movement error is large (e.g., the early part of the training trials) and slow processes, including a small forgetting and a small learning rate, are involved when the movement error is small (e.g., the latter part of the training trials). In the previous study (Ingram et al., 2013), generalization functions were investigated using error-clamp trials in which the movement error was forcibly set to 0. By contrast, for investigating context-dependent decay, error clamp trials were not used, which indicates that the movement error was not 0. If a larger number of primitives are activated in a fast process compared to a slow process, i.e., if σ is larger in the motor primitives associated with the fast process than in those associated with the slow process, the difference in the widths of the context-dependent decay and the generalization function can be reproduced. Another possible solution is to determine the recruitment pattern of motor primitives based on the movement error (Takiyama et al., 2013). In this model, the recruitment patterns of the motor primitives are different between the trials used to investigate the context-dependent decay (i.e., when the movement error is large) and the trials used to investigate the generalization function (i.e., when the movement error is small) in the experimental setting, which results in a difference in σ between the recruited primitives that enables the reproduction of the difference in the widths of the context-dependent decay and the generalization function.

### Conflict of interest statement

The authors declare that the research was conducted in the absence of any commercial or financial relationships that could be construed as a potential conflict of interest.
